# Supplementary material for: Chromothripsis during telomere crisis is independent of NHEJ, and consistent with a replicative origin
Source: Genome Res. 2019 May;29(5):737–49. doi: 10.1101/gr.240705.118 (PMC6499312; doi:10.1101/gr.240705.118)
Supplement: Supplemental Material [file supp_gr.240705.118_Supplemental_file_1.zip › contigs/annotated_contigs/DB51/contig.3.DB51_length_524_mean_cov_4.20229007634.docx]

**DB51_length_524_mean_cov_4.20229007634**

```
AGGAAACAAGAAGTTAATTTTCCTCTAGTCAATTTATCTAATTACATGTAAGTCACAACAATTTTATATATTTCTTTTCTATTTCATTT
 >chr11:91105010-91105121 - E=4e-55 p=0e+00
TTTCTCATATATATATATAT|AT|TATATATATATATATATATATATAATATTAT|ATATTA|GTATAGAGTTTTCATGGTTTCTCATG
                     >chr2:31280157-31280196 + E=8e-09 p=4e-02  >chr11:91104065-91104
ATAGGTAACATTTGCTTCAATCTAAGAACATTTATCTTTATTGAATACTACCACATGCCAGGTATTGTGCCAGCTGCTTTATCAAATTA
442 - E=2e-206
TCTCATTAATCCTTGTAGTAACTTTCCAAGTGTGTATCGTAATACTTATTTTACAAATGAATCCTCTGAAACCAGAGAGTTTAAACAGT

CAAGGTCACATACCTGGTAGGTTGTAGGACTAATACTTGAAATCACACATCTCTGTTGTTATGTAATACATTAATATAAGACTATACCT

ATTCATAGATTTGAATTCTAATCTCTCACCATTAGGGCCAAACTTGTAGAAAAAAACATAAGGTATGATATCTGCCAT|GAAAT
```
